# Moderate-to-Vigorous Physical Activity Is Associated With Cardiorespiratory Fitness Among Primary Schoolchildren Living in Côte d'Ivoire, South Africa, and Tanzania

**DOI:** 10.3389/fpubh.2021.671782

**Published:** 2021-08-19

**Authors:** Markus Gerber, Serge A. Ayekoé, Johanna Beckmann, Bassirou Bonfoh, Kouadio Benal Kouassi, Bomey Clément Gba, Sylvain G. Traoré, Jean T. Coulibaly, Dao Daouda, Rosa du Randt, Marceline F. Finda, Elihaika G. Minja, Stefanie Gall, Getrud J. Mollel, Christin Lang, Kurt Z. Long, Honorati Masanja, Ivan Müller, Siphesihle Nqweniso, Fredros O. Okumu, Nicole Probst-Hensch, Uwe Pühse, Peter Steinmann, Cheryl Walter, Jürg Utzinger

**Affiliations:** ^1^University of Basel, Basel, Switzerland; ^2^Institut National de la Jeunesse et des Sports, Abidjan, Côte d'Ivoire; ^3^Centre Suisse de Recherches Scientifiques en Côte d'Ivoire, Abidjan, Côte d'Ivoire; ^4^Unité de Formation et de Recherche des Sciences et Technologies des Aliments, Université Nangui Abrogoua, Abidjan, Côte d'Ivoire; ^5^Unité de Formation et de Recherche Biosciences, Université Félix Houphouët-Boigny, Abidjan, Côte d'Ivoire; ^6^Unité de Formation et de Recherche, Université Peleforo Gon Coulibaly, Korhogo, Côte d'Ivoire; ^7^Swiss Tropical and Public Health Institute, Basel, Switzerland; ^8^Nelson Mandela University, Gqeberha, South Africa; ^9^Ifakara Health Institute, Dar es Salaam, Tanzania

**Keywords:** accelerometry, Côte d'Ivoire, maximal oxygen uptake, sedentary behavior, South Africa, Tanzania, cardiorespiratory fitness

## Abstract

**Background:** Physical inactivity and low cardiorespiratory fitness (CRF) are independent cardiovascular risk factors among children, but have rarely been investigated concurrently in sub-Saharan Africa. The purpose of this study was to compare physical activity (PA) and CRF of primary schoolchildren living in Côte d'Ivoire (CI), South Africa (ZA), and Tanzania (TZ), to test sex- and age-related differences, and to examine whether PA and CRF are associated with each other.

**Methods:** Baseline data from an ongoing cluster-randomized controlled trial were used, including 499 children from CI (Taabo, 49% girls, *M* = 8.0 ± 1.6 years), 1,074 children from ZA (Gqeberha, 49% girls, *M* = 8.3 ± 1.4 years), and 593 children from TZ (Ifakara, 51% girls, *M* = 9.4 ± 1.7 years). PA was assessed by accelerometry and CRF by a 20 m shuttle-run test. The data were analyzed using multi-/univariate analyses of variance and mixed linear models.

**Results:** Most children met recommendations put forward by the World Health Organization for moderate-to-vigorous PA (MVPA) and achieved high CRF scores. In CI, 89.6% of the children met MVPA recommendations (boys: 91.7%, girls: 87.4%), whereas this rate was 76.9% in ZA (boys: 91.0%, girls: 62.4%), and 93.8% in TZ (boys: 95.5%, girls: 92.0%). Children from TZ had the highest CRF and MVPA levels, followed by children from CI and ZA. Boys had higher MVPA levels than girls, whereas girls engaged in more sedentary behavior. Sex differences were strongest in ZA. Sedentary behavior and MVPA were higher among older schoolchildren compared to their younger peers. Higher MVPA, but not sedentary behavior, was associated with better CRF.

**Conclusions:** In all three settings, higher levels of MVPA were associated with higher CRF scores. Nevertheless, children living in the most urbanized setting (such as observed in ZA) were physically less active and had lower CRF than peers living in more rural areas (such as observed in CI and TZ). Particularly for girls, urbanization might increase the risk for insufficient MVPA, which may have negative effects on their CRF, thus negatively influencing health and well-being at later age.

## Introduction

Opportunities for children to engage in sport, physical activity (PA), and play are regarded as a fundamental human right by the World Health Organization (WHO) and the United Nations Educational, Scientific and Cultural Organization (UNESCO) (1). Regular PA—defined as any movement of the body, which is produced by the skeletal muscles and which requires energy expenditure (e.g., at school, during leisure time, for transport) (2)—helps to ensure optimal metabolic function and to reduce the risk of chronic degenerative diseases and premature death (3). Hence, the promotion of PA among children and adolescents is given high priority in high-income countries (HICs) (4), whereas low- and middle-income countries (LMICs) have paid less attention to this issue (5, 6). Currently, international PA recommendations suggest that children and adolescents should engage in at least 60 min of moderate-to-vigorous PA (MVPA) per day (7).

Meanwhile, the positive impact of PA on children's health and well-being is well-documented across various domains, including physical, physiological, developmental, mental, cognitive, and social health, as well as academic attainment (8, 9). While evidence is largely derived from research in HICs, recent studies from LMICs confirmed the positive relationships between PA and health. For instance, a study with primary schoolchildren living in marginalized settings in the Eastern Cape province of South Africa (ZA) showed that higher PA levels are associated with higher quality of life (10), better academic achievement (11), and more favorable cardiovascular health (12), independently of whether PA is assessed via self-report (13) or accelerometer devices (14). Nevertheless, studies have also pointed toward decreasing levels of PA among children and adolescents (15). This is of particular concern, as prolonged sedentary behavior may have negative consequences for children's health (16).

In LMICs, a particularly strong decline of PA seems to occur among children living in urban settings (17). In a recent review of 298 population-based cross-sectional surveys, including 1.6 million participants, Guthold et al. (18) examined global trends in insufficient PA among youngsters aged 11–17 years. Their report suggests that globally, in 2016, 81.0% of youngsters did not engage in sufficient PA. Insufficient PA was more prevalent in girls (84.7%) than boys (77.6%). Similar findings were reported in a review that focused on youngsters aged 13–17 years from LMICs (19), in which data from 49 countries were combined and included a total of 164,771 participants. This review showed that <30% of the study population met current WHO recommendations, with boys being more likely to meet PA standards than girls. In sub-Saharan countries, the prevalence of sufficiently active youngsters varied between 8.2 and 25.6% (*M* = 15%).

Observations of lower PA in girls than boys appear consistent across sub-Saharan Africa, and in both children and adolescents (17). As highlighted by Bénéfice and Ndiayene (20, p. 367), one contributing factor is that “in traditional subsistence populations, the workload must be shared among family members, and a strict division of tasks is present according to age and sex. Children and adolescents, especially girls, are frequently involved in daily agricultural or domestic tasks. Men are generally in charge of high-energy-demanding muscular tasks, while women and children are allotted time-consuming tasks.”

With regard to age, a study showed that in children, the amount of MVPA peaks around school entry, and then starts to decline (21). Other studies, however, found that MVPA increases until the age of 12 years (22). Data from the International Children's Accelerometry Database (ICAD), including children aged between 3 and 18 years, revealed a decrease of 4.2% in total PA with each additional year of age (23). Based on a similar international cross-sectional database with 24,025 children aged 5–18 years, Corder et al. (24) observed a 6.9% relative reduction in mean vigorous PA (VPA) with every year of age (corresponding to a reduction of 7.8 min), with a stronger decline in girls (10.7%) than in boys (2.9%). For moderate PA (MPA), the reduction amounted to 6.0% (which corresponds to 22.8 min, no sex differences reported).

The assessment and interpretation of PA in children and adolescents is among the most challenging tasks in epidemiology (25). So far, self-reported PA questionnaires have been the preferred tool for global surveillance (18), but they might be susceptible to recall and response bias, have limited criterion validity, and can overestimate PA behaviors among young people (26). As a consequence, more objective measures, such as pedometers and accelerometers, are increasingly used. Accelerometers have particularly gained increasing popularity to assess habitual PA patterns in large studies because they can capture detailed and meaningful data over longer periods. Nevertheless, data on objectively measured PA levels among African primary schoolchildren are still scarce.

Despite the advantages, accelerometer application does imply some challenges: the devices are relatively expensive, including specialized software, while data collection and analyses are complex, time consuming, and permeated with compliance issues (27, 28). Hence, field-based measures of cardiorespiratory fitness (CRF) are a suitable alternative (29). CRF reflects the ability of the organism to deliver oxygen to the muscles and to utilize it for energy generation that supports muscle activity during exercise (25, 30). Peak oxygen uptake (VO_2_peak) or maximal oxygen uptake (VO_2_max) reflect the highest rate, at which oxygen can be consumed during exercise (31). Some researchers consider CRF to be a relatively stable reflection of past PA behavior, “similar to glycosylated hemoglobin, reflecting glucose control over a period of several months” [(29), p. 794].

CRF depends on a series of non-modifiable or relatively stable factors such as sex, age, growth, sexual maturation, and genetic influences. With regard to sex- and age-related differences, boys generally perform better in CRF tests than girls, by achieving higher estimated VO_2_max levels (32), independent on whether CRF is expressed in absolute (ml/min) or body mass related terms (ml/kg/min) (25). The same pattern has also been documented in children from sub-Saharan African countries (17). Nevertheless, sex differences remain relatively small until the age of 10–12 years, and then become markedly larger during adolescence (33).

Although 30–50% of CRF is determined by genetic factors, researchers assume that improvements in CRF can mainly be achieved through regular participation in PA (29). Hence, evidence suggests that trained children and adolescents have a higher VO_2_peak than untrained peers (34). Among trained boys, VO_2_peak values can reach levels of up to 60 ml/min, and among girls 50 ml/min (30). A recent meta-analysis showed that CRF is associated with children's MVPA, independent of sedentary time (35). Nevertheless, this aspect has rarely been studied in African populations. One of the few studies to address this was a cross-sectional study with 111,099 Kenyan adults (17–68 years), which yielded a significant relationship between CRF and habitual PA (*r* = 0.59) (36), yet, no such relationship was observed among Senegalese primary schoolchildren (20).

The purpose of this paper was (i) to describe and compare the PA behavior [sedentary behavior, light physical activity (LPA), MPA, VPA, and MVPA] and CRF of primary schoolchildren from three African countries [Côte d'Ivoire (CI), Tanzania (TZ), and ZA]; (ii) to examine sex- and age-related differences; and (iii) to examine whether MVPA and CRF are similarly associated with each other in boys and girls.

## Materials and Methods

### Study Design

In this paper, we present baseline data from the *KaziAfya* cluster-randomized controlled trial. In this trial, we intended to collect data at three distinct time points with primary schoolchildren from grades 1–4 from three African countries (CI, TZ, and ZA). According to the study protocol, data assessment occurred at baseline and then again at 9 and 21 months after baseline. In each participating school, we randomly assigned classes to one of four intervention conditions: (i) physical activity; (ii) multi-micronutrient supplementation; (iii) physical activity plus multi-micronutrient supplementation; and (iv) no intervention, thus serving as control. Details of the study have been presented elsewhere (37).

### Participants and Procedures

Table 1 describes the settings and periods, in/during which the baseline data assessment took place. All schools were public. We first contacted school authorities before seeking contact to schools through the school principals. Schools were eligible for the study if facilities were available for the implementation of physical education lessons and if they did not engage in any other research project or clinical trial.

**Table 1:** Overview of the locations and settings, in which the study took place in each country.

**Country**	**Côte d'Ivoire (CI)**	**South Africa (ZA)**	**Tanzania (TZ)**
Size of country (km^2^)	322,462 km^2^	1.22 million km^2^	947,303 km^2^
Number of inhabitants in country	26.45 million (2020)	57.78 million (2018)	56.31 million (2018)
Study site	The study was conducted in Taabo, which is a typical small to medium sized town located in the south-central part of Côte d'Ivoire. The Taabo site was chosen because it houses a Primary Education Inspectorate and a demographic and health surveillance system that includes 13 villages and more than 100 hamlets.	The study was conducted in the Gqeberha area, in Eastern Cape. Four schools were selected from Zwide, New Brighton, Gelvandale, and Bethelsdorp. These areas are classified as peri-urban areas.	The study was conducted in peri-urban settings in Ifakara town council in the Kilombero district in southern Tanzania. Four wards were randomly selected within Ifakara town; these were Kining'ina, Katindiuka, Mlabani, and Kibaoni.
Number of inhabitants in study site	42,480 (2014)	967,677 (Gqeberha)	The four wards have a population of between 10,000 and 20,000 people each, most of whom are subsistence rice farmers.
Number of primary schools at study site	64 (2017)	103 (quintile three schools)	Each ward has one public primary school.
Setting	Rural	Peri-urban	Peri-urban
Description of living conditions	The bulk of Taabo's economy relies on agriculture, which employs 90% of the population. It has two companies in the agricultural sector and a hydroelectric power station (hydroelectric dam) with a total energy production of 550 Mega Watt/hour (2% of the national production).	The schools are situated in historically neglected, apartheid demarcated Black African and colored areas that have been adversely affected by high unemployment rates and extreme poverty.	The majority of the residents of the town are rice farmers, but they also conduct small businesses, fishing and animal husbandry.
Number of schools taking part in study	8	4	4
Number of classes involved in study	32	41	16
Date of data assessment	October to December 2018	January to March 2019	July to August 2019

Before the start of the baseline data assessment, written informed consent was obtained from the parents/guardians of the children. Research assistants used an information sheet to explain the purpose and procedures of the study, the expected duration, potential risks and benefits, and any discomfort it may entail for the children. Additionally, all children provided oral assent before the start of the study. To be considered for data analyses, children had to attend grade 1–4; being no older than 12 years; not participating in other research projects or clinical trials; and not suffering from clinical conditions that prevented participation in PA, as determined by qualified medical personnel.

### Ethical Considerations

In CI, the study protocol was approved by the Institutional Review Board (IRB) of the Centre Suisse de Recherches Scientifiques en Côte d'Ivoire (CSRS) and the Comité National d'Ethique des Sciences de la Vie et de la Santé (CNESVS; reference number: 100–18/MSHP/CVESVS-km). In ZA, approval was granted by the research ethics committee of the Nelson Mandela University in Gqeberha (reference number: H18-HEA-HMS-006) and the Eastern Cape Departments of Education and Health. In TZ, the study protocol was approved by the responsible ethics committee at the Ifakara Health Institute (IHI–IRB; reference number: # IHI/IRB/No 39-2018), the National Institute for Medical Research (NIMR; reference number: NIMR/HQ/R.8a/Vol. IX/3137), and the Tanzania Food and Drugs Authority (TFDA; reference number: TMDA0019/CTR/0016/05). Children who suffered from severe medical conditions and/or malnourishment (as diagnosed by a nurse, following national guidelines) were referred to local clinics. Ethical approval was also obtained from the “Ethikkommission Nordwest- und Zentralschweiz” in Switzerland (EKNZ; reference number: Req-2018-00608), and the study was registered in the ISRCTN registry (http://www.isrctn.com/ISRCTN29534081).

### Assessment of Physical Activity and Cardiorespiratory Fitness

Light triaxial accelerometers (ActiGraph® wGT3X-BT; Pensacola, United States of America) were used to objectively assess children's PA behavior during one regular school week. Previous studies have shown that these monitors are a reliable measure to assess PA levels (38). The children were instructed to wear the accelerometer on 7 consecutive days around the hip, except for activities in water. A sampling rate of 30 Hz was chosen. Raw files were analyzed with the ActiLife software (version 6.13.2). A day was considered valid if children wore the monitor for at least 8 h (39). Non-wear time was estimated with default settings of the Troiano et al. (40) algorithm. Only children with valid data on ≥4 weekdays and ≥1 weekend day were included (41). The applied cut-points were specifically defined for children to calculate indices for sedentary behavior, LPA, MPA, VPA, and an overall index for MVPA (42).

CRF was assessed with the 20 m shuttle run test (43), starting with a pace of 8.5 km/h. The speed was steadily increased by 0.5 km/h, following sound signals. The test was finished when children were no longer able to follow the speed of the sound signal twice in a row. The total number of fully completed 20 m laps was noted to predict VO_2_max according to the equation put forward by Léger et al. (43). The 20 m shuttle run is a reliable and broadly validated field test to assess CRF among children (29).

### Statistical Analyses

Descriptive statistics for all study variables are reported separately for each country (Table 2). Normality was tested via the Kolmogorov-Smirnov test. If severe non-normality was observed in one country (skewness and kurtosis values of ≥ |2| and/or ≥ |7|, respectively) (44), values were log-transformed (natural log) before calculating inferential statistics. Multi- and univariate analyses of covariance ([M]ANCOVAs) were done to examine differences in PA and CRF, between countries, boys and girls, and children with different age. Bonferroni *post-hoc* tests were used to examine differences between single countries. To examine whether MVPA is associated with CRF, we carried out mixed linear regression analyses (for the total samples in each country, and separately for boys and girls). Class membership was considered as a random intercept to account for the nested nature of the data. Age, body mass index (BMI), sex, and accelerometer wear time were used as potential confounders. All statistical analyses were carried out with SPSS version 26 for Mac (IBM Corporation; Armonk, United States of America). The level of statistical significance was set at *p* < 0.05 across all analyses.

**Table 2:** Descriptive statistics for the total sample, separately for children from Côte d'Ivoire (CI), South Africa (ZA), and Tanzania (TZ).

	***M (SD)***	**Range**	**Skewness**	**Kurtosis**	**KS-test**
**CI (** ***N*** **=** **499)**					
Age (in years)	8.0 (1.6)	4.5–12.4	0.3	−0.5	*p* < 0.001
Weight (in kg)	23.4 (5.7)	12.9–65.3	2.0 (0.7)	8.8 (1.8)	*p* < 0.001
Height (in cm)	123.9 (10.4)	90–160	0.5	0.7	*p* < 0.01
BMI (kg/m^2^)	15.1 (1.5)	10.9–25.5	1.2 (0.6)	5.0 (1.9)	*p* < 0.001
**Cardiorespiratory fitness**					
20 m shuttle run (laps)	32.9 (17.6)	2–82	0.6	−0.2	*p* < 0.001
Estimated VO_2_max (in ml kg^−1^ min^−1^)	50.8 (4.9)	35.2–64.4	0.0	0.3	*p* < 0.05
**Physical activity**					
Sedentary (min/day)	570.6 (68.3)	314.5–841.7	−0.3	1.0	*p* = 0.177
LPA (min/day)	330.4 (47.2)	17.6–469.6	−0.1	0.1	*p* = 0.200^a^
MPA (min/day)	66.3 (17.5)	18.5–155.0	0.4	1.2	*p* = 0.200^a^
VPA (min/day)	26.9 (12.9)	3.7–129.5	1.8	6.6	*p* < 0.001
MVPA (min/day)	93.2 (28.3)	22.2–233.7	0.7	1.9	*p* < 0.05
**ZA (** ***N*** **=** **1,074)**					
Age (in years)	8.3 (1.4)	5.7–12.7	0.3	−0.8	*p* < 0.001
Weight (in kg)	25.3 (6.7)	13.5–61.2	1.8 (0.7)	4.7 (1.0)	*p* < 0.001
Height (in cm)	124.6 (9.0)	102–152	0.2	−0.3	*p* < 0.01
BMI (kg/m^2^)	16.1 (2.6)	20.5–29.9	2.1 (1.4)	6.1 (3.0)	*p* < 0.001
**Cardiorespiratory fitness**					
20 m shuttle run (laps)	21.9 (13.3)	3–113	1.9	5.6	*p* < 0.001
Estimated VO_2_max (in ml kg^−1^ min^−1^)	47.5 (3.8)	35.9–65.0	0.6	1.6	*p* < 0.001
**Physical activity**					
Sedentary (min/day)	609.0 (69.3)	339.8–852.3	−0.2	0.2	*p* = 0.200^a^
LPA (min/day)	325.5 (44.7)	153.3–461.9	−0.2	0.3	*p* = 0.085
MPA (min/day)	56.9 (16.8)	12.2–122.6	0.4	−0.1	*p* < 0.01
VPA (min/day)	25.2 (12.6)	3.4–115.5	1.4	4.0	*p* < 0.001
MVPA (min/day)	82.1 (27.7)	15.7–211.9	0.6	0.6	*p* < 0.001
**TZ (** ***N*** **=** **593)**					
Age (in years)	9.4 (1.7)	4.8–12.9	0.0	−0.8	*p* < 0.01
Weight (in kg)	26.5 (5.8)	143.3–49.8	0.8 (0.2)	0.8 (−0.1)	*p* < 0.001
Height (in cm)	128.3 (10.4)	100–161	0.1	−0.4	*p* = 0.200^a^
BMI (kg/m^2^)	16.0 (1.8)	11.5–29.5	1.3 (-0.2)	5.6 (0.3)	*p* < 0.001
**Cardiorespiratory fitness**					
20 m shuttle run (laps)	46.2 (18.2)	10–106	0.5	0.0	*p* < 0.001
Estimated VO_2_max (in ml kg^−1^ min^−1^)	52.0 (4.4)	36.1–63.1	−0.2	−0.1	*p* = 0.200^a^
**Physical activity**					
Sedentary (min/day)	580.6 (61.9)	394.3–798.7	0.0	−0.1	*p* = 0.200^a^
LPA (min/day)	339.0 (40.9)	196.6–475.6	0.0	−0.1	*p* = 0.200^a^
MPA (min/day)	67.5 (17.5)	24.1–130.1	0.5	0.5	*p* < 0.01
VPA (min/day)	33.3 (15.6)	6.4–147.0	1.7	6.3	*p* < 0.001
MVPA (min/day)	100.8 (30.7)	30.8–259.3	0.9	1.7	*p* < 0.001

*PA, physical activity; LPA, light-intensity physical activity; MPA, moderate-intensity physical activity; VPA, vigorous-intensity physical activity; MVPA, moderate-to-vigorous-intensity physical activity*.

a *Lower threshold of real significance*.

## Results

### Sample Characteristics

In CI, 499 children presented with complete data across all study variables (mean age: 8.0 ± 1.6 years, 252 boys, 247 girls). The mean VO_2_max was 50.8 ± 4.9 ml kg^−1^ min^−1^ and children engaged in 93.2 ± 28.3 min of MVPA per day (Table 2). Most of the variables were not normally distributed; however, severe non-normality was only observed for weight, which was then log-transformed. In total, 89.6% of the children met current MVPA standards (≥ 60 min/day).

In ZA, 1,074 children had complete data records (mean age: 8.3 ± 1.4 years, 545 boys, 529 girls). Mean VO_2_max was 47.5 ± 3.8 ml kg^−1^ min^−1^ and mean MVPA was 82.1 ± 27.7 min/day (Table 2). With the exception of sedentary behavior and LPA, none of the variables were normally distributed; severe non-normality was only observed for BMI, which disappeared after log-transformation. In total, 76.9% of the children met current MVPA standards.

In TZ, 593 children had complete data records (mean age: 9.4 ± 1.7 years, 291 boys, 302 girls). The descriptive statistics show that mean VO_2_max was 52.0 ± 4.4 ml kg^−1^ min^−1^ and children engaged in 100.8 ± 30.7 min of MVPA per day (Table 2). Although most variables were not normally distributed, we did not observe severe non-normality in any of the study variables. In total, 93.8% of the children met MVPA standards.

### Between-Country Differences

After controlling for sex, age, and BMI, MANCOVA pointed toward a significant between-country difference in CRF, Wilks-Lambda: *F*_(4, 4, 318)_ = 176.7, *p* < 0.001, η^2^ = 0.141. Univariate ANCOVAs showed that that there were significant differences between countries for laps completed in the 20 m shuttle run test, *F*_(2, 2, 160)_ = 372.4, *p* < 0.001, η^2^ = 0.256, and estimated VO_2_max, *F*_(2, 2, 160)_ = 380.1 *p* < 0.001, η^2^ = 0.260. Bonferroni *post-hoc* tests revealed significant differences between all country-pairs (*p* < 0.001). Children from TZ achieved the highest CRF levels, followed by peers from CI and ZA.

A similar pattern of findings was observed for accelerometer-based PA data. After controlling for sex, age, BMI, and accelerometer wear time, MANCOVA showed a significant main effect for country, Wilks-Lambda: *F*_(8, 4, 312)_ = 49.2, *p* < 0.001, η^2^ = 0.084. On the univariate level, ANCOVAs yielded significant between-country differences for sedentary behavior, *F*_(2, 2, 159)_ = 95.4, *p* < 0.001, η^2^ = 0.081, LPA, *F*_(2, 2, 159)_ = 51.6, *p* < 0.001, η^2^ = 0.046, MPA, *F*_(2, 2, 159)_ = 105.1, *p* < 0.001, η^2^ = 0.089, and VPA, *F*_(2, 2, 159)_ = 61.3, *p* < 0.001, η^2^ = 0.054. A significant difference was also found for MVPA, *F*_(2, 2, 159)_ = 87.1, *p* < 0.001, η^2^ = 0.075. With a few exceptions, the Bonferroni *post-hoc* tests showed significant differences between all country-pairs (*p* < 0.05). The only exceptions were that no significant differences occurred in LPA between children from CI and ZA (*p* = 0.133) and in MPA between children from CI and TZ (*p* = 0.626). Children in ZA had the highest levels of sedentary behavior, followed by peers from TZ and CI. Across all PA indicators (LPA, MPA, VPA, and MVPA), the highest values were seen in children from TZ, followed by peers from CI and ZA.

Significant between-country differences were also found in the portion of children who met MVPA standards, $\chi_{\left(2,2,166\right)}^{2}$ = 95.4, *p* < 0.001. Pair-wise comparisons showed that the portion differed between children from CI and ZA, $\chi_{\left(1,1,573\right)}^{2}$ = 35.4, *p* < 0.001, TZ and ZA, $\chi_{\left(1,1,667\right)}^{2}$ = 76.3, *p* < 0.001, and CI and TZ, $\chi_{\left(1,1,092\right)}^{2}$ = 6.3, *p* < 0.01, with the highest proportion of children meeting MVPA standards found in study participants from TZ, followed by peers from CI and ZA.

### Sex Differences

After controlling for age and BMI, MANCOVAs showed significant between-sex differences for CRF in children from ZA, Wilks-Lambda: *F*_(2, 1, 069)_ = 7.1, *p* < 0.01, η^2^ = 0.013, and TZ, Wilks-Lambda: *F*_(2, 588)_ = 63.0, *p* < 0.001, η^2^ = 0.176. By contrast, no significant between-sex differences were found in children from CI, Wilks-Lambda: *F*_(2, 494)_ = 1.7, *p* = 0.177, η^2^ = 0.007. In children from ZA and TZ, boys reached higher CRF scores than girls in the univariate analyses (Table 3).

**Table 3:** Descriptive statistics for boys and girls, separately for children from Côte d'Ivoire (CI), South Africa (ZA), and Tanzania (TZ).

	**Boys**	**Girls**			
	***M* (*SD*)**	***M* (*SD*)**	***F***	***p***	*****η^2^*****
**CI (** ***N*** **=** **499)**					
*N*	252	247			
**Cardiorespiratory fitness**					
20 m shuttle run (laps)	34.2 (17.4)	31.6 (17.8)	3.5	0.063	0.007
Estimated VO_2_max (in ml kg^−1^ min^−1^)	51.3 (4.7)	50.3 (5.1)	3.3	0.068	0.007
**Physical activity^a^**					
Sedentary (min/day)	561.6 (69.1)	579.7 (66.4)	11.6	<0.01	0.023
LPA (min/day)	331.3 (45.1)	329.5 (49.3)	0.0	0.982	0.000
MPA (min/day)	70.5 (17.9)	61.9 (16.0)	40.0	<0.001	0.075
VPA (min/day)^b^	30.4 (14.6)	23.4 (9.7)	45.5	<0.001	0.084
MVPA (min/day)	100.9 (30.1)	85.3 (23.8)	50.1	<0.001	0.092
**ZA (** ***N*** **=** **1,074)**					
*N*	545	529			
**Cardiorespiratory fitness**					
20 m shuttle run (laps)	23.4 (15.1)	20.4 (11.1)	9.2	<0.01	0.008
Estimated VO_2_max (in ml kg^−1^ min^−1^)	47.7 (4.0)	47.4 (3.6)	4.8	0.029	0.004
**Physical activity^a^**					
Sedentary (min/day)	591.0 (69.0)	627.6 (64.5)	134.2	<0.001	0.112
LPA (min/day)	331.8 (43.4)	319.1 (45.2)	31.3	<0.001	0.028
MPA (min/day)	64.4 (15.9)	49.2 (14.0)	270.7	<0.001	0.202
VPA (min/day)^b^	30.3 (13.5)	19.9 (9.1)	218.0	<0.001	0.169
MVPA (min/day)	94.6 (27.2)	69.1 (21.7)	287.2	<0.001	0.212
**TZ (** ***N*** **=** **593)**					
*N*	292	301			
**Cardiorespiratory fitness**					
20 m shuttle run (laps)	54.0 (18.6)	38.8 (14.3)	125.9	<0.001	0.176
Estimated VO_2_max (in ml kg^−1^ min^−1^)	53.6 (4.1)	50.4 (43.2)	119.6	<0.001	0.169
**Physical activity^a^**					
Sedentary (min/day)	575.3 (61.7)	585.6 (61.8)	14.4	<0.001	0.024
LPA (min/day)	335.9 (39.6)	342.1 (41.9)	2.9	0.090	0.005
MPA (min/day)	74.2 (18.3)	61.2 (13.9)	91.9	<0.001	0.135
VPA (min/day)^b^	37.9 (18.0)	28.9 (11.1)	41.5	<0.001	0.066
MVPA (min/day)	112.0 (33.4)	90.0 (23.2)	83.4	<0.001	0.124

*PA, physical activity; LPA, light-intensity physical activity; MPA, moderate-intensity physical activity; VPA, vigorous-intensity physical activity; MVPA, moderate-to-vigorous physical activity. (M)ANCOVAs are controlled for age and BMI (log transformed)*.

a *For all physical activity outcomes, wear time was considered as a covariate*.

b *If VPA was examined as an outcome variable, log-transformed scores were used*.

After controlling for age, BMI and wear time, MANCOVAs yielded significant sex differences in PA behavior in children from CI, Wilks-Lambda: *F*_(4, 492)_ = 17.8, *p* < 0.001, η^2^ = 0.098, ZA, Wilks-Lambda: *F*_(4, 1, 066)_ = 73.0, *p* < 0.001, η^2^ = 0.215, and TZ, Wilks-Lambda: *F*_(4, 585)_ = 31.6, *p* < 0.001, η^2^ = 0.178. Based on the univariate analyses (Table 3), girls reported more time for sedentary behavior, with the main effect of sex being particularly strong among children from ZA (11.2% of explained variance). With regard to MPA, VPA, and MVPA, the same pattern of results was found across all countries, with boys being more active than girls. For LPA, a significant difference was only found in children from ZA, with boys having higher scores than girls. Regarding MVPA, a particularly large main effect of sex was found in children from ZA (21.2% of explained variance), with boys engaging in ~25 min of MVPA more on average than girls.

In CI, the number of boys (91.7%) and girls (87.4%) who met the current MVPA standards was similarly high, $\chi_{\left(1,499\right)}^{2}$ = 2.4, *p* = 0.123. Likewise, no significant differences were found between boys (95.5%) and girls (92.0%) from TZ, $\chi_{\left(1,593\right)}^{2}$ = 3.1, *p* = 0.078. In ZA, however, boys (91.0%) were significantly more likely to engage in sufficient MVPA than girls (62.4%), $\chi_{\left(1,1,074\right)}^{2}$ = 123.9, *p* < 0.001.

### Age-Related Differences

**Figure 1:**
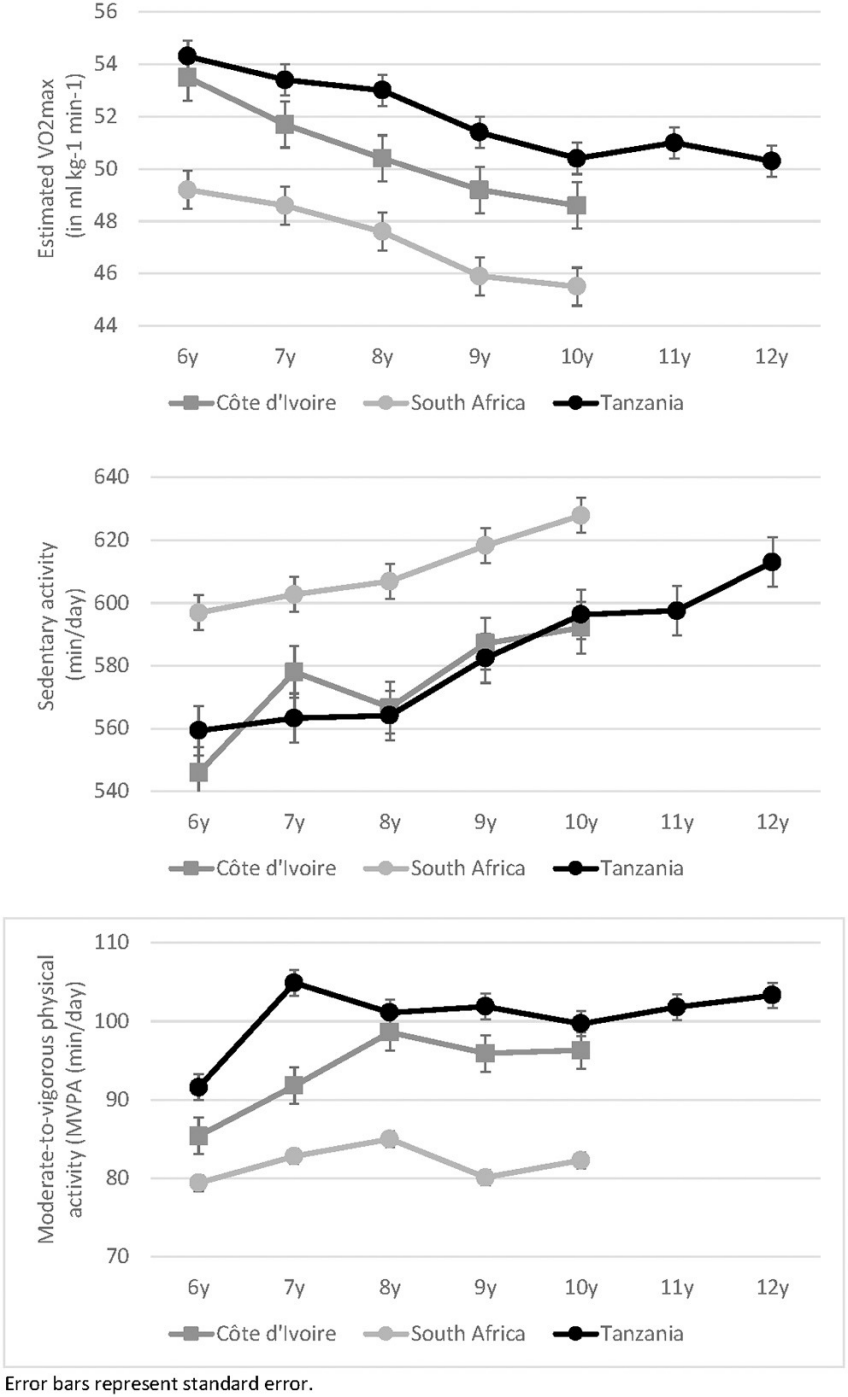
Estimated VO_2_max and moderate-to-vigorous physical activity in children with different years of age, separately for children from Côte d'Ivoire, South Africa, and Tanzania.

After controlling for sex and BMI, MANCOVAs showed that CRF differed in relation to age among children from CI, Wilks-Lambda: *F*_(8, 930)_ = 202.9, *p* < 0.001, η^2^ = 0.636, ZA, Wilks-Lambda: *F*_(8, 2, 080)_ = 7.1, *p* < 0.001, η^2^ = 0.555, and TZ, Wilks-Lambda: *F*_(12, 1, 166)_ = 223.3, *p* < 0.001, η^2^ = 0.697. As shown in Table 4, the univariate analyses revealed that the number of completed laps in the 20 m shuttle run increased from the age of 6 until the age of 10 years in all countries (total increase in CI: +23.9%, ZA: +77.7%, and TZ: +29.9%). By contrast, estimated VO_2_max decreased steadily from the age of 6 to the age of 10 years (total decrease in CI: −9.2%, ZA: −7.6%, and TZ: −7.2%). Nevertheless, in children from TZ, VO_2_max reached a plateau and no longer decreased after the age of 10 years (see Figure 1).

**Table 4:** Descriptive statistics for children with different years of age, separately for children from Côte d'Ivoire (CI), South Africa (ZA), and Tanzania (TZ).

	**6 y *M* (*SD*)**	**7 y *M* (*SD*)**	**8 y *M* (*SD*)**	**9 y *M* (*SD*)**	**10 y *M* (*SD*)**	**11 y^**c**^*M* (*SD*)**	**12 y^**c**^*M* (*SD*)**	***F***	***p***	*****η^2^*****
**CI (** ***N*** **=** **499)**										
*N*	134	99	107	77	56	16	10			
**Cardiorespiratory fitness**										
20 m shuttle run (laps)	30.5 (18.2)	31.2 (15.7)	33.9 (17.8)	34.5 (15.9)	37.8 (18.4)	—	—	2.7	0.029	0.023
Estimated VO_2_max (in ml kg^−1^ min^−1^)	53.5 (4.2)	51.7 (3.9)	50.4 (4.5)	49.2 (4.2)	48.6 (4.8)	—	—	17.4	<0.001	0.130
**Physical activity^a^**										
Sedentary (min/day)	545.9 (72.2)	578.0 (56.5)	566.7 (70.1)	587.1 (62.3)	592.1 (65.1)	—	—	4.0	<0.01	0.033
LPA (min/day)	337.6 (47.2)	344.0 (44.1)	332.9 (44.6)	317.2 (50.6)	312.1 (46.7)	—	—	11.7	<0.001	0.092
MPA (min/day)	61.9 (15.7)	65.0 (14.7)	69.5 (18.8)	67.3 (16.8)	68.3 (20.5)	—	—	2.5	0.038	0.022
VPA (min/day)^b^	23.5 (9.4)	26.8 (10.2)	29.1 (14.5)	28.6 (16.3)	27.9 (13.4)	—	—	3.5	<0.01	0.029
MVPA (min/day)	85.4 (23.8)	91.8 (23.3)	98.6 (30.9)	95.9 (30.0)	96.3 (32.2)	—	—	3.3	0.011	0.028
**ZA (** ***N*** **=** **1,074)**										
*N*	240	250	230	207	121	23	3			
**Cardiorespiratory fitness**										
20 m shuttle run (laps)	16.2 (7.1)	20.4 (11.2)	23.6 (13.7)	24.0 (15.4)	28.8 (16.9)	—	—	31.9	<0.001	0.109
Estimated VO_2_max (in ml kg^−1^ min^−1^)	49.2 (2.0)	48.6 (3.1)	47.6 (3.8)	45.9 (4.0)	45.5 (4.5)	—	—	35.2	<0.001	0.119
**Physical activity^a^**										
Sedentary (min/day)	596.9 (69.8)	602.7 (63.2)	606.9 (71.2)	618.3 (73.5)	627.9 (65.1)	—	—	8.1	<0.001	0.030
LPA (min/day)	337.3 (43.5)	329.6 (43.0)	325.4 (46.0)	318.2 (45.1)	310.6 (41.6)	—	—	15.2	<0.001	0.055
MPA (min/day)	56.1 (15.7)	57.2 (15.8)	58.0 (18.1)	55.9 (16.0)	56.9 (18.0)	—	—	1.7	0.151	0.006
VPA (min/day)^b^	23.3 (10.9)	25.6 (11.7)	27.0 (14.8)	24.2 (12.3)	25.4 (12.5)	—	—	5.7	<0.001	0.022
MVPA (min/day)	79.4 (25.5)	82.8 (25.9)	85.0 (31.1)	80.1 (27.4)	82.3 (28.5)	—	—	3.5	<0.01	0.013
**TZ (** ***N*** **=** **593)**										
*N*	63	94	95	119	113	65	44			
**Cardiorespiratory fitness**										
20 m shuttle run (laps)	36.8 (13.3)	40.1 (12.9)	45.6 (17.0)	45.3 (17.2)	47.8 (18.7)	55.8 (19.1)	58.7 (23.2)	13.6	<0.001	0.122
Estimated VO_2_max (in ml kg^−1^ min^−1^)	54.3 (3.4)	53.4 (3.1)	53.0 (3.9)	51.4 (4.3)	50.4 (4.5)	51.0 (4.8)	50.3 (5.9)	11.1	<0.001	0.102
**Physical activity^a^**										
Sedentary (min/day)	559.3 (56.5)	563.3 (62.5)	564.2 (62.6)	582.4 (59.2)	596.3 (60.9)	597.5 (53.2)	613.0 (60.2)	8.0	<0.001	0.076
LPA (min/day)	358.9 (43.9)	357.6 (42.8)	345.7 (35.6)	334.8 (36.3)	328.1 (36.7)	325.1 (36.1)	317.0 (42.9)	15.0	<0.001	0.134
MPA (min/day)	64.1 (14.4)	70.5 (16.6)	67.1 (15.2)	66.7 (18.3)	66.7 (19.1)	69.1 (19.3)	69.1 (17.9)	0.5	0.791	0.005
VPA (min/day)^b^	27.6 (10.1)	34.4 (16.8)	34.0 (13.7)	35.1 (17.9)	33.0 (14.2)	32.7 (15.6)	34.2 (18.3)	1.9	0.072	0.020
MVPA (min/day)	91.6 (22.8)	104.9 (32.2)	101.1 (26.0)	101.9 (33.5)	99.7 (31.4)	101.8 (33.2)	103.3 (34.3)	1.0	0.424	0.010

*PA, physical activity; LPA, light-intensity physical activity; MPA, moderate-intensity physical activity; VPA, vigorous-intensity physical activity; MVPA, moderate-to-vigorous physical activity. (M)ANCOVAs are controlled for sex and BMI (log transformed)*.

a *For all physical activity outcomes, wear time was considered as a covariate*.

b *If VPA was examined as an outcome variable, log-transformed scores were used*.

c *In the (M)ANCOVAs, 11- and 12-year-old children were only considered in the TZ sample, because in the samples from CI the number of children aged 11 or 12 years were low (N ≤ 25 children per year of age category)*.

After controlling for age, BMI, and wear time, MANCOVAs yielded significant age-related differences in PA behavior in children from CI, Wilks-Lambda: *F*_(16, 1, 412)_ = 5.7, *p* < 0.001, η^2^ = 0.047, ZA, Wilks-Lambda: *F*_(16, 3, 169)_ = 6.7, *p* < 0.001, η^2^ = 0.025, and TZ, Wilks-Lambda: *F*_(24, 2, 025)_ = 4.9, *p* = 0.001, η^2^ = 0.048. More specifically, the univariate analyses (Table 4) showed that sedentary behavior significantly increased in all countries from the age of 6–10 years (total increase in CI: +8.5%, ZA: +5.2%, and TZ: +6.6%; see Figure 1). However, older children had higher MVPA scores than younger peers, a finding that was observed across all countries (total increase from age 6 to 10 years in CI: +12.8%, ZA: +3.7%, and TZ: +8.8%; see Figure 1).

Regarding the proportion of children who met MVPA standards, we did not observe significant age-related differences in any of the countries [CI, $\chi_{\left(4,473\right)}^{2}$ = 8.7, *p* = 0.68, ZA, $\chi_{\left(4,1,048\right)}^{2}$ = 3.9, *p* = 0.415, and TZ, $\chi_{\left(6,593\right)}^{2}$ = 8.2, *p* = 0.223].

### Association Between Physical Activity and Cardiorespiratory Fitness

Table 5 shows the results of the mixed linear regression models, first for the total sample in each country, then separately for girls and boys. In Table 5, sedentary behavior and MVPA are used as independent predictors. In the total sample, age was negatively associated with estimated VO_2_max in all countries. In children from ZA and TZ, higher BMI scores were associated with lower VO_2_max. Moreover, sex differences were confirmed in the regression models in TZ children. A statistically significant (positive) relationship was found between MVPA and VO_2_max in children from CI (*B* = 0.03, *p* < 0.05, 95% confidence interval = 0.01–0.05), ZA (*B* = 0.04, *p* < 0.001, 95% confidence interval = 0.02–0.05); and TZ (*B* = 0.03, *p* < 0.01, 95% confidence interval = 0.01–0.04). Table 5 further shows that whereas higher levels of MVPA were consistently associated with higher VO_2_max in girls from all three countries, in boys, a significant relationship (*p* < 0.001) was only found in ZA schoolchildren, although results pointed to the same direction in children from CI and TZ.

**Table 5:** Association between children's physical activity and cardiorespiratory fitness in Côte d'Ivoire (CI), South Africa (ZA), and Tanzania (TZ).

**Total sample**						
	**Prediction of estimated VO**_****2****_ **max with MVPA**					
	**CI (** ***N*** **=** **499)**		**ZA (** ***N*** **=** **1,074)**		**TZ (** ***N*** **=** **593)**	
	***B***	**95% confidence interval**	***B***	**95% confidence interval**	***B***	**95% confidence interval**
Age	−1.43^***^	−1.70 to −1.16	−1.06^***^	−1.21 to −0.91	−0.77^***^	−0.97 to −0.59
Sex	−0.39	−1.20 to 0.42	0.25	−0.19 to 0.69	−2.86^***^	−3.52 to −2.20
BMI (log)	−7.71	−17.34 to 1.92	−10.81^***^	−14.08 to −7.55	−11.29^**^	−17.85 to −4.72
Wear time	−0.01	−0.01 to 0.01	−0.00	−0.01 to 0.00	0.00	−0.01 to 0.01
Sedentary activity	0.01	−0.01 to 0.02	0.00	−0.01 to 0.01	0.00	−0.01 to 0.01
MVPA	0.03^*^	0.01 to 0.05	0.04^***^	0.02 to 0.05	0.03^**^	0.01 to 0.04
**Girls**						
	**Prediction of estimated VO**_**2**_ **max with MVPA**					
	**CI (** ***N*** **=** **247)**		**ZA (** ***N*** **=** **529)**		**TZ (** ***N*** **=** **301)**	
	***B***	**95% confidence interval**	***B***	**95% confidence interval**	***B***	**95% confidence interval**
Age	−1.48^***^	−1.88 to 1.09	−1.29^***^	−1.47 to −1.08	−1.19^***^	−1.45 to −0.92
BMI (log)	−10.39	−22.85 to 2.07	−10.92^***^	−14.80 to −7.04	−6.74	−15.08 to 1.61
Wear time	0.00	−0.02 to 0.01	0.00	−0.01 to 0.01	0.00	−0.02 to 0.01
Sedentary activity	0.01	−0.01 to 0.02	0.00	−0.01 to 0.01	0.00	−0.01 to 0.01
MVPA	0.04^*^	0.00 to 0.07	0.04^***^	0.02 to 0.05	0.04^***^	0.02 to 0.07
**Boys**						
	**Prediction of estimated VO**_**2**_ **max with MVPA**					
	**CI (** ***N*** **=** **252)**		**ZA (** ***N*** **=** **545)**		**TZ (** ***N*** **=** **292)**	
	***B***	**95% confidence interval**	***B***	**95% confidence interval**	***B***	**95% confidence interval**
Age	−1.35^***^	−1.73 to−0.97	−0.88^***^	−1.11 to −0.66	−0.39^**^	−0.08 to −0.11
BMI (log)	−2.19	−18.12 to 13.75	−10.95^***^	−16.49 to −5.41	−13.63^*^	−23.95 to −3.31
Wear time	−0.01	−0.02 to 0.01	0.00	−0.01 to 0.01	0.00	−0.01 to 0.02
Sedentary activity	0.00	−0.01 to 0.02	0.00	−0.1 to 0.01	−0.01	−0.02 to 0.01
MVPA	0.02	−0.01 to 0.05	0.03^***^	0.02 to 0.05	0.02	−0.01 to 0.04

*MVPA, moderate-to-vigorous physical activity*.

* *p < 0.05*.

** *p < 0.01*.

*** *p < 0.001*.

**Figure 2:**
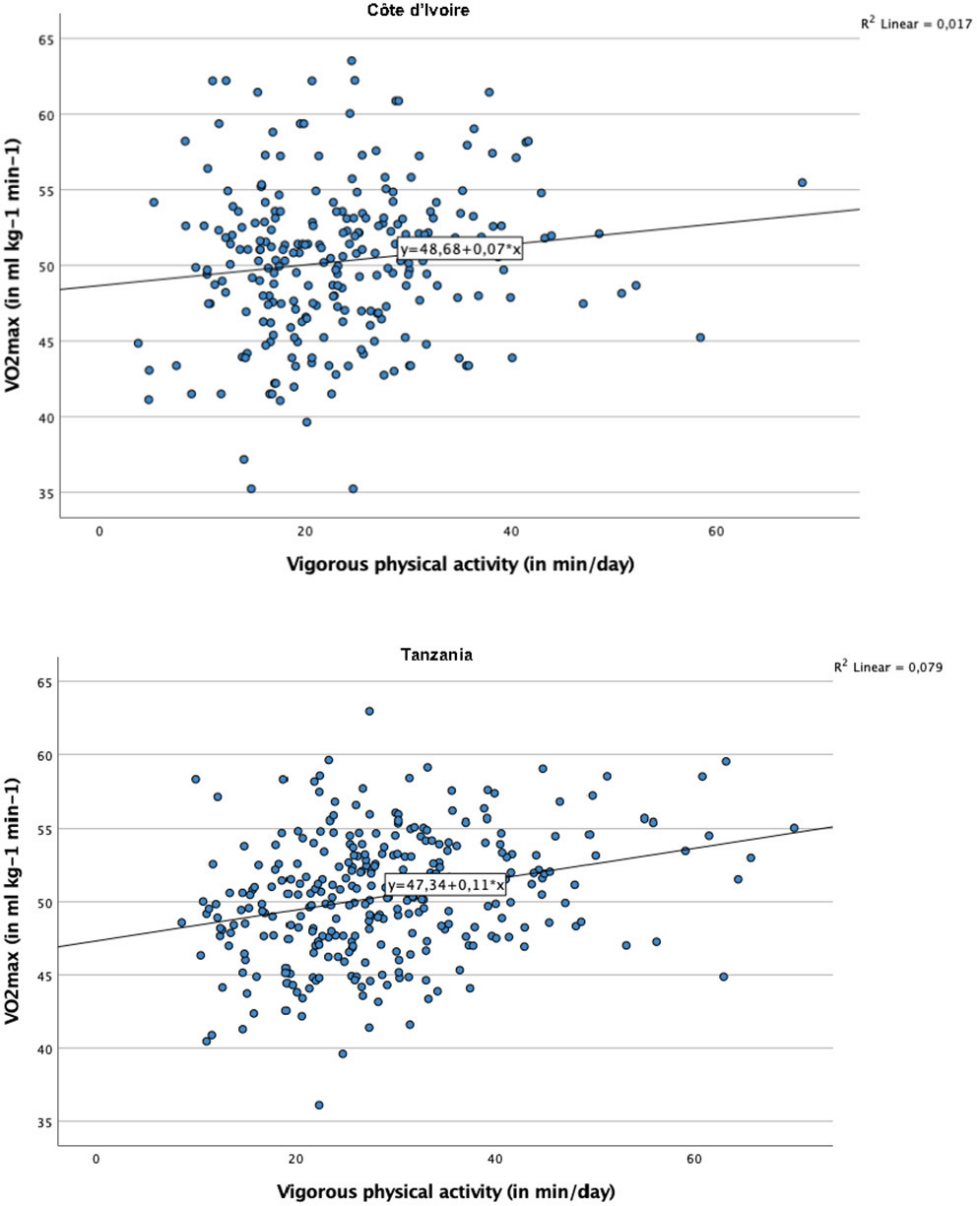
Scatter plot with regression line representing the association between vigorous physical activity and cardiorespiratory fitness, for girls from Côte d'Ivoire and Tanzania.

**Figure 3:**
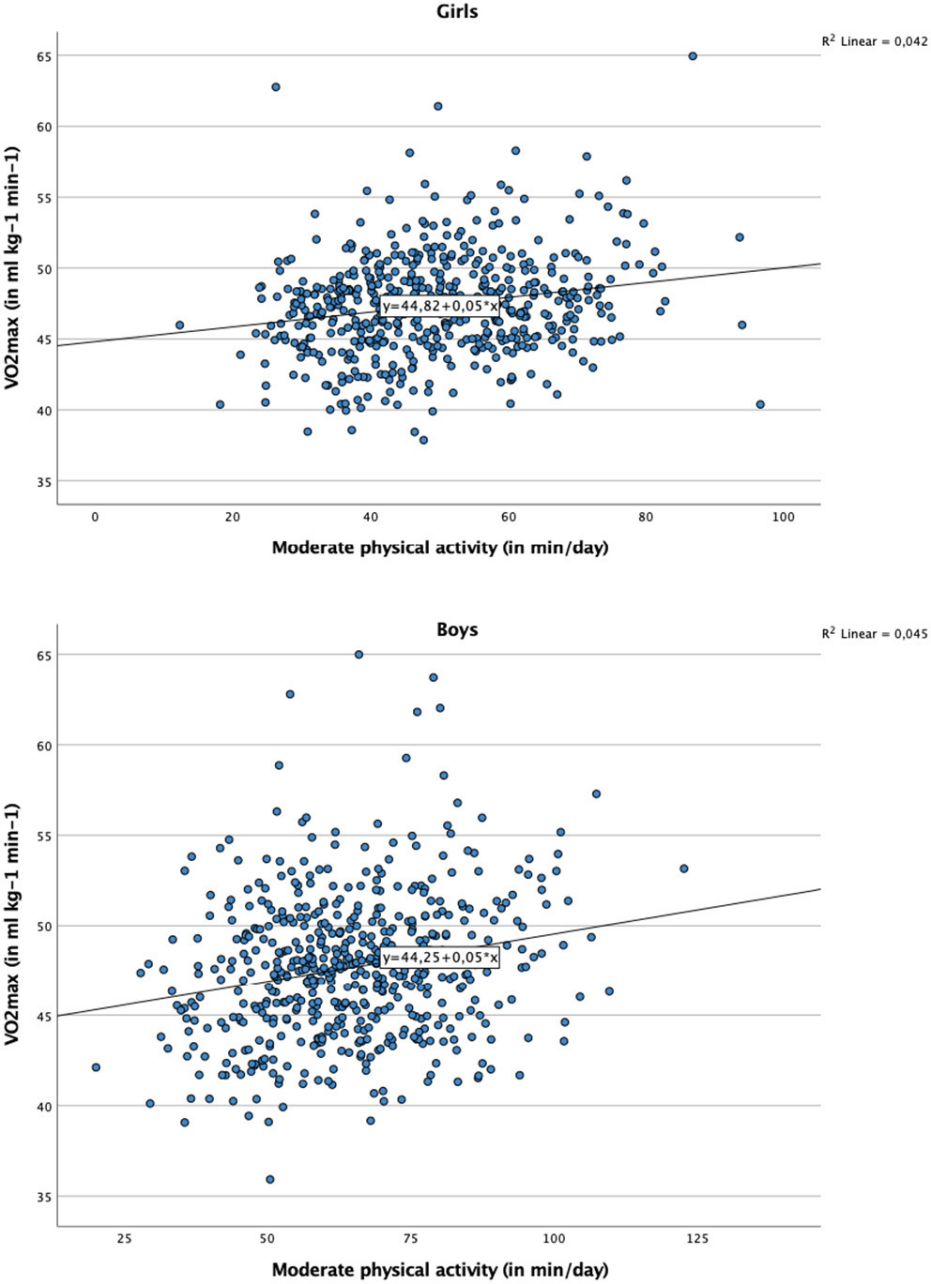
Scatter plot with regression line representing the association between moderate physical activity and cardiorespiratory fitness, for girls and boys from South Africa.

Table 6 makes a further distinction between LPA, MPA, and VPA in the prediction of CRF (in addition to sedentary behavior). In children from CI and TZ, a significant association with VO_2_max was observed for VPA (CI: *B* = 0.05, *p* < 0.05, 95% confidence interval = 0.01–0.10; TZ: *B* = 0.05, *p* < 0.01, 95% confidence interval = 0.01–0.08), but not for LPA and MPA. Nevertheless, this relationship only existed in girls (see scatterplots in Figure 2), whereas among boys no significant association between VPA and CRF occurred. In children from ZA, only MPA (but not LPA and VPA) was associated with VO_2_max. This association was found both in girls (*B* = 0.04, *p* < 0.05, 95% confidence interval = 0.01–0.07) and boys (*B* = 0.05, *p* < 0.01, 95% confidence interval = 0.01–0.08) (scatterplots shown in Figure 3).

**Table 6:** Association between physical activities with different intensities and children's cardiorespiratory fitness in Côte d'Ivoire (CI), South Africa (ZA), and Tanzania (TZ).

**Total sample**						
	**Prediction of estimated VO**_****2****_ **max with MVPA**					
	**CI (** ***N*** **=** **499)**		**ZA (** ***N*** **=** **1,074)**		**TZ (** ***N*** **=** **593)**	
	***B***	**95% confidence interval**	***B***	**95% confidence interval**	***B***	**95% confidence interval**
Age	−1.40^***^	−1.67 to −1.12	−1.07^***^	−1.21 to −0.92	−0.76^***^	−0.96 to −0.56
Sex	−0.38	−1.19 to 0.42	0.26	−0.18 to 0.71	−2.95^***^	−3.61 to −2.28
BMI (log)	−7.09	−16.74 to 2.56	−10.84^***^	−14.11 to −7.58	−10.98^**^	−17.56 to −4.41
Wear time^a^	—	—	—	—	—	—
Sedentary activity	0.00	−0.01 to 0.01	0.00	−0.01 to 0.01	0.00	−0.01 to 0.01
LPA	−0.00	−0.01 to 0.01	0.00	−0.01 to 0.01	0.00	−0.01 to 0.01
MPA	−0.01	−0.05 to 0.03	0.04^***^	0.02 to 0.07	0.01	−0.02 to 0.04
VPA	0.05^*^	0.01 to 0.10	0.02	−0.01 to 0.05	0.05^**^	0.01 to 0.08
**Girls**						
	**Prediction of estimated VO**_**2**_ **max with MVPA**					
	**CI (** ***N*** **=** **247)**		**ZA (** ***N*** **=** **529)**		**TZ (** ***N*** **=** **301)**	
	***B***	**95% confidence interval**	***B***	**95% confidence interval**	***B***	**95% confidence interval**
Age	−1.41^***^	−1.81 to −1.01	−1.27^***^	−1.47 to −1.08	−1.16^***^	−1.43 to −0.90
BMI (log)	−8.79	−21.32 to 3.73	−10.92^***^	−14.81 to −7.04	−6.28	−14.60 to 2.04
Wear time^a^	—	—	—	—	—	—
Sedentary activity	0.01	−0.01 to 0.02	0.00	−0.01 to 0.01	0.00	−0.01 to 0.01
LPA	0.00	−0.01 to 0.02	0.00	−0.01 to 0.01	0.00	−0.01 to 0.01
MPA	−0.02	−0.08 to 0.04	0.04^*^	0.01 to 0.07	0.00	−0.05 to 0.05
VPA	0.11^*^	0.02 to 0.19	0.03	−0.02 to 0.07	0.09^**^	0.04 to 0.14
**Boys**						
	**Prediction of estimated VO**_**2**_ **max with MVPA**					
	**CI (** ***N*** **=** **252)**		**SA (** ***N*** **=** **545)**		**TZ (** ***N*** **=** **292)**	
	***B***	**95% confidence interval**	***B***	**95% confidence interval**	***B***	**95% confidence interval**
Age	−1.32^***^	−1.71 to −0.94	−0.89^***^	−1.11 to −0.67	−0.38^**^	−0.67 to −0.10
BMI (log)	−2.02	−17.97 to 13.94	−11.08^***^	−16.63 to −5.53	−13.31^*^	−23.68 to −2.95
Wear time^a^	—	—	—	—	—	—
Sedentary activity	0.00	−0.01 to 0.01	0.00	−0.01 to 0.01	0.00	−0.01 to 0.01
LPA	0.00	−0.02 to 0.01	−0.01	−0.01 to 0.00	0.01	−0.01 to 0.02
MPA	−0.01	−0.06 to 0.05	0.05^**^	0.01 to 0.08	0.00	−0.04 to 0.04
VPA	0.03	−0.02 to 0.09	0.02	−0.02 to 0.05	0.03	−0.01 to 0.07

*LPA, light physical activity; MPA, moderate physical activity; VPA, vigorous physical activity*.

a *Wear time was not included as a covariate because wear time corresponds to the sum of sedentary activity, LPA, MPA, and VPA*.

* *p < 0.05*.

** *p < 0.01*.

*** *p < 0.001*.

## Discussion

The key findings of this study are that across the three African countries, most children met current MVPA recommendations and achieved relatively high CRF levels. Between-country comparisons revealed that children from TZ had the highest CRF and MVPA levels, followed by children from CI and peers from ZA. Boys had consistently higher PA levels than girls, whereas girls engaged in more sedentary behavior than boys. In two countries (ZA and TZ), boys also had higher CRF levels than girls. Absolute performance in a 20 m shuttle run (completed laps) increased with age, but estimated VO_2_max was decreasing with age. Both sedentary behavior and time spent in MVPA were higher among older children compared to their younger peers. MVPA was (positively) associated with estimated VO_2_max in the three study countries, whereas sedentary behavior was not.

With the present paper, we address four specific objectives. Firstly, we gained a deeper understanding of PA behavior and CRF of primary schoolchildren from three African countries. Our findings are at odds with previous reports showing that in sub-Saharan Africa, the majority of young people fail to meet MVPA standards (18, 19). However, previous reports focused on older children and adolescents (≥11 years) and relied on self-reports, whereas in our study, we examined younger, pre-adolescent children (≤12 years) and used accelerometer devices to objectively assess PA. This approach provides new insights into children‘s actual PA levels from African countries, and thus fills an important gap, as only few studies used accelerometer devices thus far (14). The fact that in our study the lowest PA levels were found among children from ZA (living in the most urbanized setting) was not a surprise, given that in LMICs, PA levels have more strongly declined in urban settings compared to rural settings (17), where schoolchildren often still walk relatively long distances to and from school. Researchers have emphasized that living in urban settings might significantly limit the opportunities for children to engage in regular and sufficient PA (45). Contributing factors are safety concerns that make it difficult for children to walk to schools or to parks, financial constraints that limit parents' possibilities to let their children participate in organized sport activities, general lack of sport facilities, and lacking facilities and equipment at schools (46).

With regard to children's CRF, Ruiz et al. (47) concluded that the CRF cut-points to avoid cardiovascular disease risk are 41.8 ml kg^−1^ min^−1^ for boys and 34.6 ml kg^−1^ min^−1^ for girls. In our study, the mean scores ranged from 47.7 to 53.6 ml kg^−1^ min^−1^ in boys and from 47.4 to 50.4 ml kg^−1^ min^−1^ in girls, which highlights that CRF was relatively high in the present sample. Studies have pointed toward considerable between-country difference in the 20 m shuttle run test, which might be reflective of variations in the socioeconomic, cultural, and political circumstances in the different countries (33, 48). Lang et al. (48) synthesized studies on shuttle run performance from 50 countries with children and adolescents aged 9–17 years (1,142,026 participants). The review showed that best performances in the 20 m shuttle run were observed among youth from TZ (97th percentile rank). Performances of children and adolescents from CI were still relatively good (75th percentile rank), whereas those of youth from ZA were average (54th percentile rank), ranking similarly as countries like Spain or Switzerland.

The second objective of our paper was to examine whether PA and CRF differ between boys and girls. The findings of the present study accord well with previous investigations (17), indicating that boys engage in more MVPA than girls. However, only in the ZA sample, a large gap between the two sexes was observed, with significantly fewer girls meeting MVPA standards than their male peers. This observation might indicate that urbanization and the changing life styles increase sex differences in PA. Adebusoye et al. [(49), p. 554] argued that urban development in Africa might reduce the number of conducive spaces for outdoor PA, which might be even less accessible for girls. Given that the highest population growth (and especially growth in urban areas) is expected over the next few decades in Africa and Asia (50), it is important to understand the factors that influence children's PA behaviors in these regions to create effective interventions to prevent a decline in PA (51). In this respect, in urbanized settings, girls constitute a special target group as they are more likely to engage in insufficient MVPA.

Our findings across all three countries further corroborate that boys reach higher CRF levels than girls (17, 25, 32). However, these differences were negligible in younger children from ZA and CI, a finding that confirms previous research (33). Age might also explain why the observed sex differences were larger among children from TZ, as the mean age was higher among children from TZ compared to peers from the other participating countries (this is due to the different average age at the start of school). Researchers have suggested that higher VO_2_peak scores are largely due to greater stroke volume in boys (52). Some researchers have further claimed that differences in cardiac size and cardiac function might be additional contributing factors (53). However, a study with prepubertal children using thoratic bioimpedance and cardiac magnetic resonance imaging showed that VO_2_peak did not depend on maximal stroke volume or resting heart size, but seemed to depend on children's maximal arterio-venous oxygen differences. Other researchers claimed that sex differences in VO_2_peak might be a result of poorer matching of muscle oxygen delivery to oxygen utilization in girls, while others have suggested that the differences might be due to the fact that boys are more physically active (54).

The third objective of our research was to determine whether CRF and PA differ between children according to age. In our study, sedentary behavior and MVPA were higher in older children compared to their younger peers. This is in line with previous studies showing that MVPA peaks around the age of 12 years (22). From a public health perspective, it is problematic that sedentary behavior increases with age. For instance, in ZA, recent Healthy Active Kids South Africa (HAKSA) report cards reported that many children and adolescents engage in very high levels of sedentary behavior (55, 56). On average, children watched almost 3 h of TV per day, with 31% of girls and 25% of boys watching TV more than 5 h during a normal school day (57).

Our results conflict with prior laboratory-based research showing that VO_2_peak almost linearly increases in children between the age of 8 and 14 years (30). Nevertheless, our results support data from a cross-sectional survey with 156 schoolchildren aged 7–15 years from CI, in which VO_2_max decreased with age (32). The reason for these diverging results can probably be attributed to methodological issues. For instance, Armstrong and Welsman (58) warned against an overinterpretation of CRF scores derived from the 20 m shuttle run test. First, they argued that expressing VO_2_peak in relation to age might be problematic, as peak oxygen uptake increases in accordance with morphological and physiological changes associated with growth and maturation and as there are substantial inter-individual differences with regard to the timing and tempo of these changes. Moreover, limited common variance was observed between performances in the 20 m shuttle run test and rigorously determined peak oxygen uptake in the laboratory among 11- to 14-year-old British schoolchildren (59). On the other hand, the 20 m shuttle run test is currently regarded as the most adequate field-based measure of CRF in children and youth due to its low costs, simplicity (no need for specialized human resources, easily interpretable results), and ability to assess large groups of participants at the same time (48, 60). Moreover, this test proved to be a relevant marker of children's current (61) and future cardiovascular health (62).

The fourth objective of our study was to investigate whether MVPA is associated with CRF among schoolchildren. Our findings support this notion, which is in line with a recent meta-analysis, showing that irrespective of time spent in sedentary activities, CRF and MVPA are positively associated in children (35). Our study also corroborates field-based studies in which a significant association was found between objectively assessed MVPA and children's CRF (63, 64). The findings of our mixed linear regression analyses show that the mean VO_2_max score increased by 0.03–0.04 ml kg^−1^ min^−1^ per additional min/day of MVPA. Nevertheless, it should be noted that in children from CI and TZ, the association between MVPA and CRF was only significant for girls. Furthermore, among girls from CI and TZ, CRF was only associated with VPA, but not with MPA. This contrasts with findings observed in our ZA sample, in which MPA were more closely associated with children's CRF. The reason why VPA was not significantly associated with CRF in children from ZA is not entirely clear. It is conceivable that the generally high MVPA and CRF levels (particularly among boys) may have complicated the detection of significant association between these variables (due to limited inter-individual variation).

The strengths of our study were that PA and CRF were assessed objectively, data from three different countries were considered, analyses were controlled for potential confounders (sex, age, BMI, and wear time), and mixed linear models accounted for the nested nature of the data. Moreover, although we had to exclude a substantial number of learners due to missing data and although we were not able to achieve similar sample sizes in each study site, the final samples were still relatively large (between 499 and 1,074 children per country). Future studies should include longitudinal data to gain further insights about cause and effect. We do acknowledge that the samples of the three study sites are not representative for their country, which complicates between-country comparisons, as multiple (unassessed) factors might have influenced the findings. For instance, although children were assessed from settings that were classified as “peri-urban” in each country, “peri-urban” might refer to different standards in the three countries. Another limitation is a potential selection bias as schools (clusters) were selected considering logistical/operational reasons and not truly at random. Lastly, we acknowledge that in the present analyses, we did not consider socioeconomic status as a potential confounder. Such analyses are currently underway, and will be presented elsewhere.

## Conclusions

Most children involved in our study from three African countries met current PA standards (above 75%) and achieved high CRF levels. Although the samples are not representative for the three countries included here, we observed that children living in the most urbanized setting (ZA) were physically less active and had lower CRF than peers living in more rural areas (from CI and TZ). Because PA differences were particularly found between boys and girls who live in the most urbanized setting, our findings suggest that urbanization might increase the risk for girls to not engage in sufficient MVPA. Therefore, girls should be seen as a particularly relevant target group for health interventions, especially as regular PA might contribute to the maintenance of good CRF, with positive implications for health and well-being in older age.

## Data Availability Statement

The raw data supporting the conclusions of this article will be made available by the authors, without undue reservation.

## Ethics Statement

The studies involving human participants were reviewed and approved by In Côte d'Ivoire, the study protocol was approved by the Institutional Review Board (IRB) of the Centre Suisse de Recherches Scientifiques en Côte d'Ivoire (CSRS; Abidjan, Côte d'Ivoire) and the Comité National d'Ethique des Sciences de la Vie et de la Santé (CNESVS; reference number: 100–18/MSHP/CVESVS-km). In South Africa, approval was granted by the research ethics committee of the Nelson Mandela University in Gqeberha (reference number: H18-HEA-HMS-006) and the Eastern Cape Departments of Education and Health. In Tanzania, the study protocol was approved by the responsible ethics committee at the Ifakara Health Institute (IHI–IRB; reference number: # IHI/IRB/No 39-2018), the National Institute for Medical Research (NIMR; reference number: NIMR/HQ/R.8a/Vol. IX/3137) and the Tanzania Food and Drugs Authority (TFDA; reference number: TMDA0019/CTR/0016/05). Children who suffered from severe medical conditions and/or malnourishment (as diagnosed by a nurse, following national guidelines) were referred to local clinics. Ethical approval was also obtained from the Ethikkommission Nordwest- und Zentralschweiz in Switzerland (EKNZ; reference number: Req-2018-00608), and the intervention study was registered in the ISRCTN registry (http://www.isrctn.com/ISRCTN29534081). Written informed consent to participate in this study was provided by the participants' legal guardian/next of kin.

## Author Contributions

MG, KL, UP, and JU designed the study. MG serves as principal investigator of the study, performed the statistical analysis, and wrote the first draft of the manuscript. BB, HM, and CW are the principal investigators in the three partner countries. CL is responsible for the overall coordination of the study. ST, MF, and SN are the local coordinators. RdR, DD, KL, FO, NP-H, UP, and PS served as project advisors. SA, JB, BK, BG, ST, JC, MF, EM, SG, GM, CL, IM, and SN contributed to the data assessment and processing. All authors contributed to manuscript revision, read, and approved the submitted version.

## Conflict of Interest

The authors declare that the research was conducted in the absence of any commercial or financial relationships that could be construed as a potential conflict of interest.

## Publisher's Note

All claims expressed in this article are solely those of the authors and do not necessarily represent those of their affiliated organizations, or those of the publisher, the editors and the reviewers. Any product that may be evaluated in this article, or claim that may be made by its manufacturer, is not guaranteed or endorsed by the publisher.

## Abbreviations

BMI: Body mass index
CI: Côte d'Ivoire
CNESVS: Comité National d'Ethique des Sciences de la Vie et de la Santé
CRF: Cardiorespiratory fitness
CSRS: Centre Suisse de Recherches Scientifiques en Côte d'Ivoire
EKNZ: Ethikkommission Nordwest- und Zentralschweiz' in Switzerland
HAKSA: Healthy Active Kids South Africa
HICs: High income countries
ICAD: International Children's Accelerometry Database
IHI: Ifakara Health Institute
IRB: Institutional Review Board
ISRCTN: International Standardized Randomized Controlled Trial Number
LMICs: Low- and middle income countries
LPA: Light-intensity physical activity
(M)ANCOVA: (Multivariate) analyse of covariance
MPA: Moderate-intensity physical activity
MVPA: Moderate-to-vigorous physical activity
NIMR: National Institute for Medical Research
PA: Physical activity
TFDA: Tanzania Food and Drugs Authority
TV: Television
TZ: Tanzania
UNESCO: United Nations Educational, Scientific and Cultural Organization
VO_2_max: Maximal oxygen uptake
VO_2_peak: Peak oxygen uptake
VPA: Vigorous-intensity physical activity
WHO: World Health Organization
ZA: South Africa.
